# Long COVID-19 syndrome associated with Omicron XBB.1.5 infection: a case report

**DOI:** 10.1590/0074-02760230069

**Published:** 2023-10-13

**Authors:** Otávio Espíndola, Paola C Resende, Lusiele Guaraldo, Guilherme Amaral Calvet, Trevon L Fuller, Stephanie Lema Suarez Penetra, Heloisa Ferreira Pinto Santos, Anielle Pina-Costa, Michele Fernanda Borges da Silva, Isabella Campos Vargas Moraes, Fernando Medeiros, Jimmy Whitworth, Christopher Smith, Karin Nielsen-Saines, Marilda M Siqueira, Patrícia Brasil

**Affiliations:** 1 Fundação Oswaldo Cruz-Fiocruz, Instituto Nacional de Infectologia Evandro Chagas, Laboratório de Pesquisa Clínica em Doenças Febris Agudas, Rio de Janeiro, RJ, Brasil; 2 Fundação Oswaldo Cruz-Fiocruz, Instituto Oswaldo Cruz, Laboratório de Vírus Respiratórios e do Sarampo, Rio de Janeiro, RJ, Brasil; 3 University of California Los Angeles, Institute of the Environment and Sustainability, Los Angeles, USA; 4 London School of Hygiene and Tropical Medicine, Department of Clinical Research, London, UK; 5 Nagasaki University, School of Tropical Medicine and Global Health, Nagasaki, Japan; 6 University of California Los Angeles, David Geffen School of Medicine, Division of Pediatric Infectious Diseases, Los Angeles, USA

**Keywords:** SARS-CoV-2, post-acute COVID-19 syndrome, immune evasion

## Abstract

**BACKGROUND:**

There is interest in lingering non-specific symptoms after severe acute respiratory syndrome coronavirus 2 (SARS-CoV-2) infection, referred to as Long coronavirus disease 2019 (Long COVID-19). It remains unknown whether the risk of Long COVID-19 is associated with pre-existing comorbidities or initial COVID-19 severity, including infections due to new Omicron lineages which predominated in 2023.

**OBJECTIVES:**

The aim of this case report was to characterize the clinical features of acute XBB.1.5 infection followed by Long COVID-19.

**METHODS:**

We followed a 73-year old female resident of Rio de Janeiro with laboratory-confirmed SARS-CoV-2 during acute infection and subsequent months. The SARS-CoV-2 lineage was determined by genome sequencing.

**FINDINGS:**

The participant denied comorbidities and had completed a two-dose vaccination schedule followed by two booster doses eight months prior to SARS-CoV-2 infection. Primary infection by viral lineage XBB.1.5. was clinically mild, but the participant subsequently reported persistent fatigue.

**MAIN CONCLUSIONS:**

This case demonstrates that Long COVID-19 may develop even after mild disease due to SARS-CoV-2 in fully vaccinated and boosted individuals without comorbidities. Continued monitoring of new SARS-CoV-2 lineages and associated clinical outcomes is warranted. Measures to prevent infection should continue to be implemented including development of new vaccines and antivirals effective against novel variants.

The emergence of successive severe acute respiratory syndrome coronavirus 2 (SARS-CoV-2) variants has been a cause for concern among surveillance networks and the scientific community in general, as new variants have the potential to evade SARS-CoV-2 immunity conferred by vaccination and/or infection. The Omicron variant of concern (VOC) became predominant globally in the third quarter of 2021. Subsequently, successive Omicron lineages arose and became predominant. During the first half of 2023, the Omicron lineage XBB.1.5 was predominant in the United States and Europe.^1^ ^,^ ^2^ XBB.1.5 arose through the recombination of previous Omicron lineages and carries the F486P spike mutation, which confers higher affinity to the angiotensin-converting enzyme 2 (ACE2) receptor.^3^ XBB.1.5 was first detected in Brazil in the State of São Paulo, and on January 6, 2023 in the city of Rio de Janeiro.

In addition to the public health burden of acute Coronavirus disease 2019 (COVID-19), there is now considerable interest in conditions involving clusters of non-specific symptoms that linger after primary infection with SARS-CoV-2, referred to as Long COVID-19. A variety of partially overlapping definitions of Long COVID-19 were developed by the United States Centers for Disease Control (CDC), United Kingdom Research and Innovation (UKRI), and the World Health Organization (WHO). These definitions included clusters of symptoms such as breathlessness, cognitive impairment (“brain fog”, difficulty in thinking, poor attention, memory loss, and confusion), fatigue, orthostatic intolerance, post-exertional malaise, and sleep disorders.^4^ ^,^ ^5^ ^,^ ^6^ In the researching COVID to enhance recovery (RECOVER) cohort with 9,764 participants, the prevalence of Long COVID-19 was 10% six months after infection.^7^ However, prevalence estimates of Long COVID-19 have varied among studies. According to a recent review, several mechanisms have been proposed to explain the Long COVID-19 phenomenon, including organ damage, viral persistence, reactivation of Epstein-Barr virus, inflammatory activation, endothelial dysfunction, mast cell activation, autoimmune disorders, and dysbiosis of the microbiome.^8^ The objective of this case report was to characterize the clinical features of acute XBB.1.5 infection followed by Long COVID-19.

## SUBJECTS AND METHODS

We followed a patient identified in Rio de Janeiro, a 73-year-old woman who had been in contact with asymptomatic visitors from New York City, NY, USA, since December 24, 2022. She had received four vaccine doses: full vaccination with ChAdOx1 (AstraZeneca) on April 21, 2021, a BNT162b2 (Pfizer-BioNTech) booster dose on October 2, 2021, and a second booster with Ad26.COV2.S (Janssen) on May 03, 2022, eight months before infection. On January 2, 2023, she displayed mild COVID-19 symptoms, including fatigue, sore throat, and nasal congestion, followed by cough, nausea, and sleep disturbances. No fever, ageusia/anosmia, paresthesia, myalgia, arthropathy, headache, cognitive and memory alterations, nor ocular, skin, bladder, or intestinal manifestations were observed. She did not receive any specific treatment as nirmatrelvir/ritonavir is not readily available in Brazil. Fatigue persisted for over a month, characterizing Long COVID-19 syndrome. Red and white blood cell counts and biomarkers of hepatic function, including aspartate and alanine aminotransferases, gamma glutamyl transferase, alkaline phosphatase, and bilirubin were normal throughout the entire follow-up period. One month post-infection, ferritin, C reactive protein, creatinine, creatine phosphokinase, albumin, serum total proteins, amylase, and lipase were all normal. SARS-CoV-2 infection was confirmed by real time reverse transcription polymerase chain reaction (RT-PCR) in the naso/oropharyngeal swabs and saliva, with a cycle threshold (Ct) of 19.44 and 20.94, respectively, for the nucleoprotein gene. The genomic sequences described in the study were deposited in the Global Initiative on Sharing All Influenza Data (GISAID) database (accession numbers below).

*Ethics* - Study procedures were in accordance with the Declaration of Helsinki of 1975, as revised in 1983, and approved by the Brazilian National Council of Ethics in Research (CONEP) (protocol 30639420.0.0000.5262). Informed consent was obtained from the subject involved in the study.

## RESULTS

XBB.1.5 was identified by SARS-CoV-2 whole genome sequencing (EPI_ISL_16741399). The Table shows the frequency of Omicron lineages in Brazil over time. According to the AudacityInstant (v5.0.1) GISAID tool (www.gisaid.org), the genomes most closely related to the genome detected in this study were from North America. Viral load declined after one week (Ct 32.29 for the naso/oropharyngeal swabs and 33.63 for saliva) becoming undetectable one month later. The patient had no previous history of SARS-CoV-2 infection, confirmed by a negative serum nucleoprotein anti-SARS-CoV-2 Immunoglobulin G (IgG). Following an eight-month interval since the last administration of a SARS-CoV-2 vaccine, only low IgG anti-Spike Protein S1 levels (3,633 AU/mL) were detected at the time of infection.

**TABLE:** Frequency of Omicron lineages in Brazil from November 1, 2022 to March 31, 2023

Lineage	Month of collection									
	November 2022		December 2022		January 2023		February 2023		March 2023	
	N	%	N	%	N	%	N	%	N	%
BA.1/BA.1.*	6	0.1%	3	0.1%	4	0.3%	1	0.2%		0%
BA.2/BA.2.*	18	0.2%	7	0.1%	8	0.6%	5	1.1%	1	0.6%
BA.3	1	0.01%	1	0.02%	1	0.1%		0%		0%
BE.9	531	6.9%	431	8.4%	123	8.6%	29	6.5%		0%
BE.10	1024	13.2%	416	8.1%	57	4.0%	5	1.1%		0%
BQ.1/BQ.1.*	4416	57.1%	3685	71.4%	957	66.6%	80	17.9%	4	2.2%
DL.1	1318	17%	242	4.7%	20	1.4%	6	1.3%		0%
Other	295	3.8%	166	3.2%	63	4.4%	14	3.1%	2	1.1%
Other XBB*	123	1.6%	180	3.5%	128	8.9%	140	31.3%	90	50.3%
XBB.1.5	1	0.01%	29	0.6%	77	5.4%	167	37.4%	82	45.8%

*descendants of the Pango lineage.

## DISCUSSION

This case has bearing upon a number of unresolved questions related to Long COVID-19. Firstly, it demonstrates that there is the possibility of occurrence of Long COVID-19 even after mild acute infection in a patient without any comorbidities. A previous study described a case of mild acute SARS-CoV-2 infection followed by chronic fatigue syndrome, with prolonged loss of taste, and impaired capillary microcirculation in the macula and peripapillary region in a patient with a documented history of glaucoma.^9^ Unlike the previous study, our case did not have any comorbidities before SARS-CoV-2 infection. It remains unknown whether the risk of Long COVID-19 is associated with comorbidities or the severity of the initial COVID-19 infection. Secondly, Long COVID-19 occurred in a fully vaccinated and boosted individual, demonstrating that even mild infection may be associated with Long COVID-19.

XBB.1.5 appears to elicit lower neutralizing antibody titers than previous Omicron lineages, even among individuals who are vaccinated or possess hybrid immunity (infection combined with vaccination).^10^ Although the spread of XBB.1.5 did not appear to increase COVID-19 hospitalizations,^11^ whether XBB.1.5 and any other new Omicron variant are associated with the development of a higher frequency of Long COVID-19 cases remains unknown.

As in many other countries, XBB.1.5 emerged as the most prevalent SARS-CoV-2 lineage in Brazil during the first half of 2023. At a time when decision makers are considering reducing resources for COVID-19 surveillance, such emergence underscores the need for continued monitoring of new lineages and implementing measures to prevent infection such as the development of new vaccines and antivirals effective against a novel variant.
